# SARS-CoV envelope protein palmitoylation or nucleocapid association is not required for promoting virus-like particle production

**DOI:** 10.1186/1423-0127-21-34

**Published:** 2014-04-27

**Authors:** Ying-Tzu Tseng, Shiu-Mei Wang, Kuo-Jung Huang, Chin-Tien Wang

**Affiliations:** 1 Department of Medical Research, Taipei Veterans General Hospital, 201, Sec. 2, Shih-Pai Road, Taipei 11217, Taiwan; 2 Institute of Clinical Medicine, National Yang-Ming University School of Medicine, 201, Sec. 2, Shih-Pai Road, Taipei 11217, Taiwan

## Abstract

**Background:**

Coronavirus membrane (M) proteins are capable of interacting with nucleocapsid (N) and envelope (E) proteins. Severe acute respiratory syndrome coronavirus (SARS-CoV) M co-expression with either N or E is sufficient for producing virus-like particles (VLPs), although at a lower level compared to M, N and E co-expression. Whether E can release from cells or E/N interaction exists so as to contribute to enhanced VLP production is unknown. It also remains to be determined whether E palmitoylation or disulfide bond formation plays a role in SARS-CoV virus assembly.

**Results:**

SARS-CoV N is released from cells through an association with E protein-containing vesicles. Further analysis suggests that domains involved in E/N interaction are largely located in both carboxyl-terminal regions. Changing all three E cysteine residues to alanines did not exert negative effects on E release, E association with N, or E enhancement of VLP production, suggesting that E palmitoylation modification or disulfide bond formation is not required for SARS-CoV virus assembly. We found that removal of the last E carboxyl-terminal residue markedly affected E release, N association, and VLP incorporation, but did not significantly compromise the contribution of E to efficient VLP production.

**Conclusions:**

The independence of the SARS-CoV E enhancement effect on VLP production from its viral packaging capacity suggests a distinct SARS-CoV E role in virus assembly.

## Background

Coronaviruses are enveloped viruses with 27–32 kb single-strand positive-sense RNA genomes encoding four structural proteins: nucleocapsid (N), spike (S), membrane (M) and envelope (E) [1,2]. Translated on free polysomes, highly basic N interacts with newly synthesized viral genomic RNA to form helical nucleocapsids [3,4]. The M, S and E viral membrane proteins are translated on membrane-bound polysomes, inserted into the endoplasmic reticulum (ER), and transported to the ER-Golgi intermediate compartment (ERGIC), where E and M interact and trigger budding [5,6]. N and S are incorporated into virions via interaction with M, with virions accumulating in large, smooth-walled vesicles that eventually fuse with the plasma membrane and release virions from cells [2,7-11].

Coronavirus E is a small integral membrane protein consisting of approximately 76 to 109 amino acids and containing a hydrophobic domain. Several researchers have suggested that coronavirus E functions as an ion channel [12,13]. The role of the coronavirus E ion channel in the virus life cycle is not completely clear. The addition of hexamethylene amiloride (HMA, an ion channel inhibitor of mouse hepatitis coronavirus [MHV] and human coronavirus 229E [HCoV229E] ion channel activity *in vitro*) to culture medium blocks MHV and HCoV229E replication [12], suggesting that the coronavirus E ion channel plays a role in virus replication. Two or three cysteine residues are located on the carboxyl side of the hydrophobic domain in coronavirus E proteins, with some serving as targets for palmitoylation [14-17], which may contribute to virus assembly in the MHV.

E plus M has been shown to be sufficient for VLP formation in the MHV [18], transmissible gastroenteritis virus (TGEV) [19], bovine coronavirus (BCoV) [19], infectious bronchitis virus (IBV) [5], and SARS-CoV [20]. Although M and N co-expression is also sufficient for SARS-CoV VLP production [21,22], VLP yields are further enhanced by E co-expression [23]. According to these data, SARS-CoV E plays a supporting role in promoting virus assembly and/or budding. E and N are thought to be packaged into VLPs through separate and individual associations with M; it remains unknown whether E/N interaction exists, which might contribute to enhanced virion production. MHV and IBV E proteins are capable of release from cells as vesicles [5,24], implying a relationship between E release and its contribution to virus production. Our goal for this study was to determine whether SARS-CoV N is capable of interacting with E and cell release via an association with E-containing vesicles. Our results indicate no correlation between SARS-CoV E capacity to release or interact with N, or its ability to promote VLP production.

## Methods

### Plasmid construction

Mammalian expression vectors encoding SARS-CoV M, N, S and E were provided by G. J. Nabel [21]. A pair of upstream and downstream primers was used to amplify E-coding fragments via PCR-based overlap extension mutagenesis [25]. Primers used to introduce an HA or FLAG epitope tag to the E amino or carboxyl terminus are 5′-TTCTGCGATATCGCCACCATGTACCCATACGACGTGCCTGACTACGCCTACAGCTTCGTGAGCG-3′ (containing a flanking EcoRV restriction site and HA tag-coding nucleotides) and 5′-GCGGATCCTCACTTGTCGTCGTCGTCCTTGTAGTCGCCCACCAGCAGGTCGGGCAC-3′ (containing a flanking BamHI restriction site and FLAG tag-coding nucleotides). The forward primer is 5′-GTCTGAGCAGTACTCGTTGCTG-3 (referred to as the N primer) and reverse primer 5′-GGAAAGGACAGTGGGAGTGGCAC-3′ (referred to as the anti-N primer). Primers used to construct designated E mutants were EC3A, 5′- CTGAGGCTGGCCGCATATGCCGCGAACATCGTGAACGTGAGC-3′ (reverse); 74LL/AA,5′-GCAGATCTGGATCCTAGTTCACACGGCCGCGTCGGGCACGCC-3′ (reverse); EΔ76V, 5′- CAGATCTGGATCCTAGTTCACAGCAGGTCGGGCAC-3′ (reverse). The N primer serves as a forward primer while the anti-N primer was used as a reverse primer for the second round PCR amplification. Purified PCR product was digested with BamHI and EcoRV and ligated into the SARS-CoV E expression vector. When constructing N-DsRed, the N primer served as the forward primer, using the SARS-CoV N expression vector as a template and 5′- GCGGATCCTGGGTGCTGTCGGCGCTG-3′ as the reverse primer. Amplified PCR product was digested with BamHI and SalI and ligated into pDsRed-Monomer-N1 (Clontech). The cloned N-DsRed was digested with NheI and BamHI and ligated into pEGFP-N1 (Clonetech), yielding construct N-EGFP.

GST-M was constructed by digesting M expression vector with EcoRV and NotI, ligated into pCDNA3.1myc-HisA (Invitrogen). The resultant construct was then digested with BamHI and Not1, and then the M coding sequence fragment was fused to carboxyl terminus of GST, which is directed by a mammalian elongation factor Ia promoter [26]. GST-N fusions, as described previously [27], were constructed by fusing SARS-CoV N coding sequences to the carboxyl terminus of GST.

### Cell culture and transfection

293 T and HeLa cells were maintained in Dulbecco’s modified Eagle’s medium (DMEM) supplemented with 10% fetal calf serum (GIBCO). Confluent cells were trypsinized and split 1:10 onto 10 cm dishes 24 h prior to transfection. For each construct, cells were transfected with 20 μg of plasmid DNA using the calcium phosphate precipitation method; 50 μm chloroquine was added to enhance transfection efficiency. Unless otherwise indicated, 10 μg of each plasmid was used for co-transfection. Culture supernatant and cells were harvested for protein analysis 2–3 d post-transfection. For HeLa transfection, plasmid DNA was mixed with GenCarrier (Epoch Biolabs) at a ratio of 1 μg to 1 μl; the transfection procedure was performed according to the manufacturer’s protocols.

### Western immunoblot

At 48–72 h post-transfection, supernatant from transfected cells was collected, filtered, and centrifuged through 2 ml of 20% sucrose in TSE (10 mM Tris–HCl [pH 7.5], 100 mM NaCl, 1 mM EDTA plus 0.1 mM phenylmethylsulfonyl fluoride [PMSF]) at 4°C for 40 min at 274,000 x *g*. Pellets were suspended in IPB (20 mM Tris–HCl [pH 7.5], 150 mM NaCl, 1 mM EDTA, 0.1% SDS, 0.5% sodium deoxycholate, 1% Triton X-100, 0.02% sodium azide) plus 0.1 mM PMSF. Cells were rinsed with ice-cold phosphate-buffered saline (PBS), collected in IPB plus 0.1 mM PMSF, and microcentrifuged at 4°C for 15 min at 13,700 x *g* to remove unbroken cells and debris. Supernatant and cell samples were mixed with equal volumes of 2X sample buffer (12.5 mM Tris–HCl [pH 6.8], 2% SDS, 20% glycerol, 0.25% bromphenol blue) and 5% β-mercaptoethanol and boiled for 5 min or (for the M-containing samples) incubated at 45°C for 10 min. Samples were resolved by electrophoresis on SDS-polyacrylamide gels and electroblotted onto nitrocellulose membranes. Membrane-bound M, M-FLAG, HA-E, E-FLAG or GST proteins were immunodetected using a SARS-CoV M rabbit anitserum, anti-HA (LTK BioLaboratories, Taiwan), anti-FLAG or anti-GST(Sigma) monoclonal antibody at a dilution of 1:1,000. For SARS-CoV N or S detection, a mouse monoclonal antibody [28,29] was used at a dilution of 1:5,000. The secondary antibody was a sheep anti-mouse or donkey anti-rabbit horseradish peroxidase-(HRP) conjugated antibody (Invitrogen), both at 1:5,000 dilutions.

### Laser scanning immunofluorescence microscopy

HeLa cells were split 1:80 onto coverslips 24 h before transfection. Between 18 and 24 h post-transfection, , cells were washed with PBS and permeabilized at room temperature for 10 min in PBS plus 0.1% Triton X-100 following fixation at 4°C for 20 min with methanol/acetone (1:1). Samples were incubated with the primary antibody for 1 h and with the secondary antibody for 30 min. Following each incubation, samples were subjected to three washes (5 to 10 min each) with DMEM/calf serum. Primary antibody concentrations were anti-HA at a dilution of 1:500. A rabbit anti-mouse rhodamine-conjugated antibody at a 1:100 dilution served as the secondary antibody (Cappel, ICN Pharmaceuticals, Aurora, OH). After a final DMEM/calf serum wash, the coverslips were washed three times with PBS and mounted in 50% glycerol in PBS for viewing. Images were analyzed and photographs taken using the inverted laser Zeiss.

### Iodixanol density gradient fractionation

Supernatants from transfected 293 T cells were collected, filtered, and centrifuged through 2 ml 20% sucrose cushions as described above. Viral pellets were suspended in PBS buffer and laid on top of a pre-made 10-40% iodixanol (OptiPrep) gradient consisting of 1.25 ml layers of 10, 20, 30 and 40% iodixanol solution prepared according to the manufacturer’s instructions (Axis-Shield, Norway). Gradients were centrifuged in a SW50.1 rotor at 40,000 rpm for 16 h at 4°C; 500 μl fractions were collected from top to bottom and densities were measured for each. Proteins in each fraction were precipitated with 10% trichloroacetic acid (TCA) and subjected to Western immunoblotting.

### GST pull-down assay

GST pull-down protocols were as previously described [27]. Briefly, 500 μl of PNS containing complete protease inhibitor cocktail was mixed with 30 μl of glutathione agarose beads (Sigma). All reactions took place at 4°C overnight on a rocking mixer. Immunoprecipitate-associated resin or bead-bound complexes were pelleted, washed tree times with lysis buffer, two times with PBS, eluted with 1X sample buffer, and subjected to SDS-10% PAGE as described above.

## Results

### SARS-CoV E is capable of associating with N

**Figure 1:**
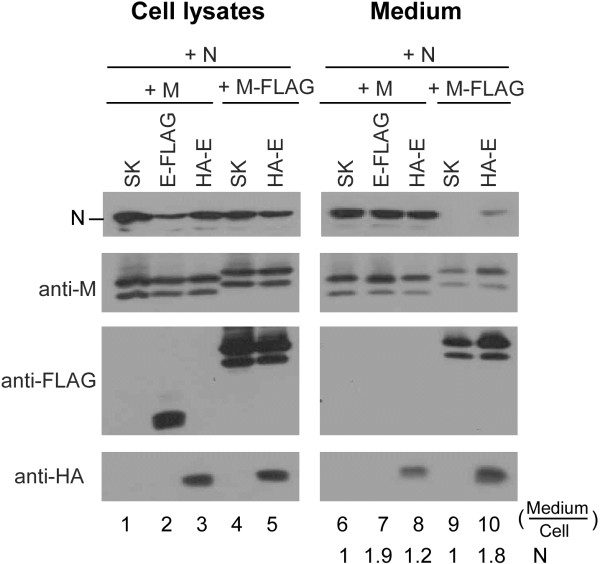
**SARS-CoV VLP assembly and release.** 293 T cells were co-transfected with SARS-CoV N, M and E expression vectors in various combinations. M-FLAG or E-FLAG indicates a FLAG tagged at the SARS-CoV M or E carboxyl terminus; HA-E denotes an HA tagged at the SARS-CoV E amino terminus. DNA (5 μg) of each plasmid was used for each transfection, with pBlueScript SK added to maintain a plasmid DNA level of 20 μg. At 48 h post-transfection, supernatants and cells were collected and prepared for protein analysis as described in Materials and Methods. Cell lysate samples (lanes 1 to 5) corresponding to 5% of total, and medium pellet samples (lanes 6 to 10) corresponding to 50% of total, were fractionated by 10% SDS-PAGE and electroblotted onto nitrocellulose filters. SARS-CoV M was probed with rabbit antiserum. SARS-CoV N and E were detected with a mouse anti-N, anti-HA, or anti-FLAG monoclonal antibody. N proteins from medium or cell samples were quantified by scanning N band densities from immunoblots. Rations of N level in media to those in cells were determined for each samples and normalized to that of samples without HA-E or E-FLAG co-expression.

In a previous study we found that SARS-CoV M co-expression with N leads to VLP formation, and that a FLAG tagged at the SARS-CoV M carboxyl terminus (M-FLAG) significantly inhibits N packaging into VLPs [22]. Since N release into medium depends on an association with M, and since E may promote VLP production, we set out to confirm whether E complements M-FLAG in N packaging into VLPs. Toward this goal we co-expressed N with M-FLAG in the absence or presence of SARS-CoV E tagged with either HA at the amino terminus (HA-E) or with FLAG at the carboxyl terminus (E-FLAG). As shown in Figure 1, N that was barely detectable in medium became readily detected following co-expression with HA-E (lanes 9 versus 10). Since N is incapable of associating with M-FLAG, a simple explanation would be the ability of E to promote N incorporation into VLPs via E/N interaction. E-FLAG was barely detected in medium (Figure 1, lane 7), suggesting that the FLAG tagged at the E carboxyl terminus affected E release or E association with M and/or N. To confirm that E-mediated N release is not due to HA tag, we co-expressed N with E and found that E without HA tag can still promote N release (data not shown), suggesting that HA tag exerts no major impacts on E-mediated N release.

**Figure 2:**
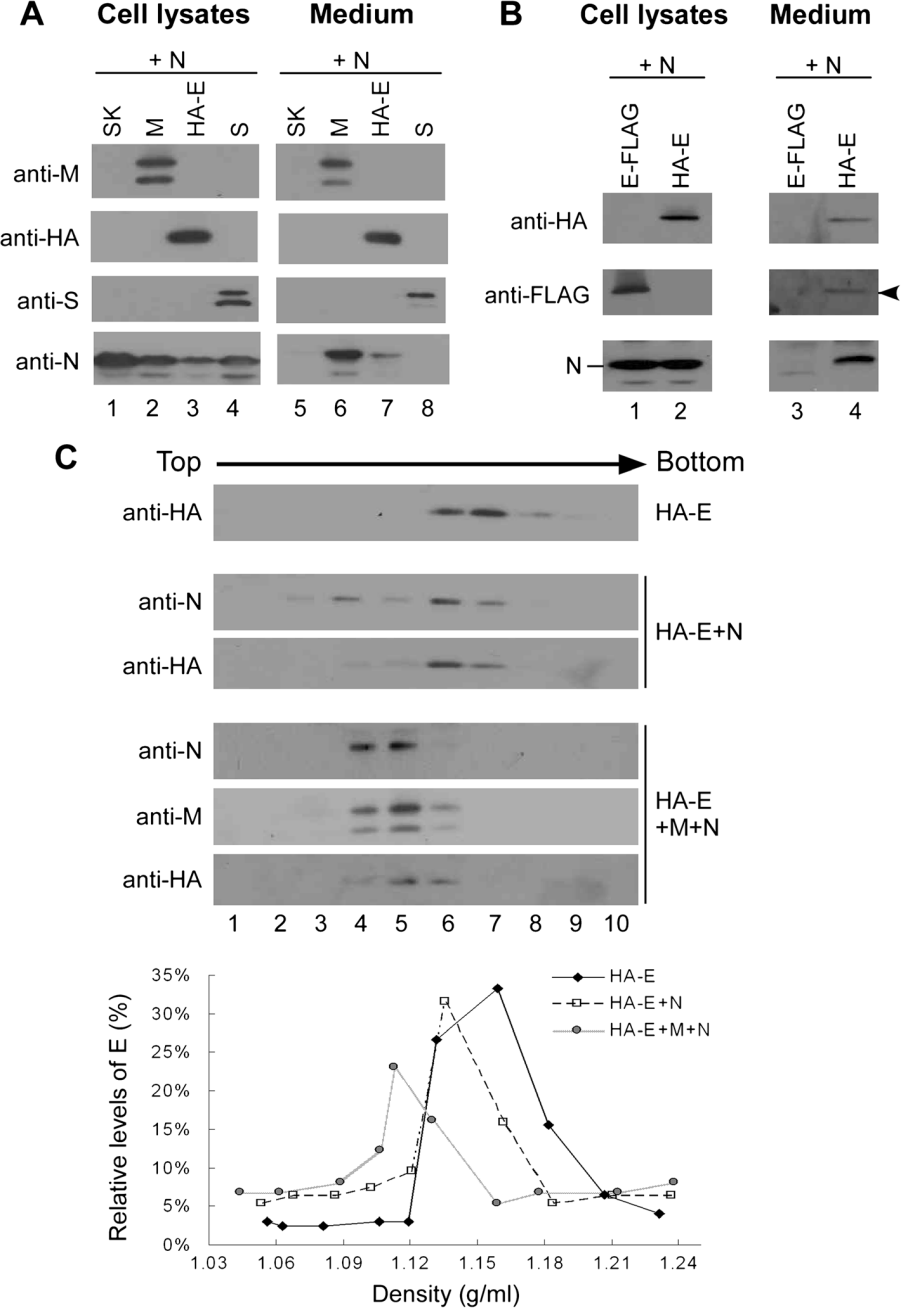
**SARS-CoV VLP analysis. (A-B)** 293 T cells were co-transfected with E tagged with either a FLAG or HA and N, M or S expression vectors. Culture supernatants were collected 48 h post-transfection and subjected to Western immunoblotting. E proteins tagged with HA were probed with an anti-HA antibody. The lane 4 band (panel **B**, arrowhead) resulted from the incomplete stripping of an anti-HA probe. **(C)** For buoyant density gradient analysis, concentrated supernatants derived from E, E plus N, or E plus M and N transfection samples were centrifuged through a 10-40% iodixanol gradient for 16 h. Equal quantities of ten fractions were collected from top to bottom. Fraction densities were measured and SARS-CoV, E, M and N proteins analyzed by Western immunoblotting, using anti-HA, anti-M and anti-N antibodies as probes.

Since SARS-CoV E and M are both capable of release into medium, N detected in medium may be a result of an association with released M-FLAG in the presence of E, but not a result of direct E/N interaction. To rule out this possibility and to confirm that a specific N-E interaction exists, we co-expressed N and either HA-E, E-FLAG, M or S. As expected, N was readily detected when co-expressed with HA-E or with M, but barely detectable in culture medium when expressed alone or co-expressed with S (Figure 2A, lanes 5 and 8) or E-FLAG (which was also barely detectable in medium) (Figure 2B, lane 3). These data support our hypothesis that SARS-CoV N is capable of release into medium via association with E, and suggests that FLAG tagged at the SARS-CoV E carboxyl terminus significantly reduces E release capacity in addition to impairing E incorporation into VLPs.

To confirm that N release is associated with E in pelletable particle form, we subjected culture medium from cells expressing E, E plus N, or E plus N and M to iodixanol density gradient fractionation, and found that E primarily sedimented with co-expressed N at a slightly lower density fraction compared to E expression alone (Figure 2C). Particles formed by E or E plus N exhibited an iodixanol density of 1.13 to 1.16 g/ml, whereas VLPs formed by E, N and M displayed densities ranging from 1.11 to 1.13 g/ml. Combined, these data support the idea that N released into medium is associated with E vesicles when co-expressed with E.

### RNA is not required for efficient E/N interaction

**Figure 3:**
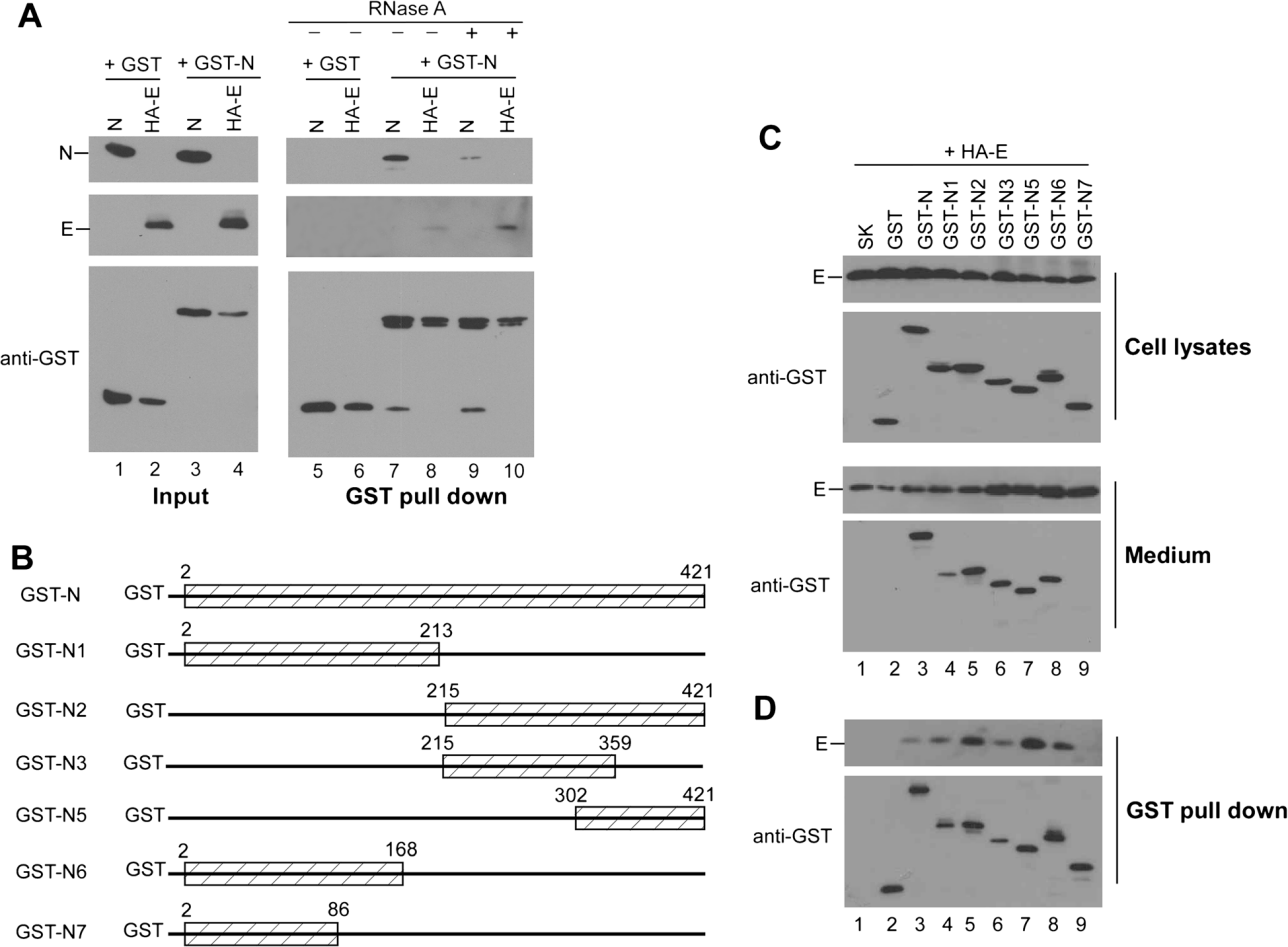
**Analysis of E/N association. (A)** E/N association is RNA independent. 293 T cells were co-transfected with the designated constructs. At 48 h post-transfection, equal amounts of the cell lysates were treated with or without 0.2 mg/ml DNase-free RNase A for 30 min at 25°C followed by mixing with glutathione-agarose beads. Complexes bound to beads were pelleted, washed, and subjected to Western immunoblotting. **(B-D)** Mapping of SARS-CoV N domains involved in E association. **(B)** Schematic representation of GST-N fusion constructs. PCR-amplified fragments containing various portions of SARS-CoV N coding sequences were fused to the carboxyl terminus of GST, directed by a mammalian elongation factor 1a promoter. **(C)** Association of GST-N fusion proteins with SARS-CoV E. 293 T cells were co-transfected with SARS-CoV HA-E and indicated GST-N fusion construct. Cells and supernatants were collected at 48 h post-transfection, prepared, and subjected to Western immunoblot analysis. **(D)** Co-precipitation of SARS-CoV E with GST-N fusion proteins. Equivalent amounts of cell lysates were analyzed by GST pull down assays as described above.

SARS-CoV N contains an RNA binding domain [30], and results from our previous study indicate that RNA can enhance the N-N interaction [22]. Here our goal was to determine whether the presence of RNA is required for E/N interaction. HA-E or N was co-expressed with GST tagged at the N amino terminus (GST-N). E or N association with GST-N was assessed using a GST pull-down assay in the presence or absence of RNase. GST by itself was not capable of pulling down HA-E or N (Figure 3A, lanes 5–6). RNase treatment significantly reduced levels of co-pulled-down N (Figure 3A, lane 9), which is consistent with past results [22]. However, the same RNase condition did not reduce the amount of co-pulled-down HA-E, suggesting that RNA is not required for E/N interaction. We frequently observed slight increases in pulled-down HA-E following treatment with RNase (Figure 3A, lane 10 vs. lane 8). Since RNA binds to N readily, it is likely that RNA-bound N molecules may associate less efficiently with E.

### E association domain is largely located in the N carboxyl-terminal region

To map the N sequences involved in E association, we constructed a set of GST-N fusions containing multiple N coding sequences fused to the GST carboxyl terminus (Figure 3B). Each GST fusion construct was transiently co-expressed with E, and culture supernatants and cell lysates were analyzed by Western immunoblotting. As shown in Figure 3C, all GST fusions except for GST-N7 (containing N residues 2 to 86, lane 9) were released into medium when co-expressed with E. None of the GST fusions were readily detected in medium without co-expressed E (data not shown), suggesting that the detected GST-N fusions were released into medium via E association, and that the E binding domains are largely located in the N carboxyl-terminal region (likely involving amino acid residues 87 to 421). Results from a GST pull-down assay (Figure 3D) provide further support for this conclusion.

### Cysteine residues are not required for E release, E/N interaction, or E enhancement of VLP production

**Figure 4:**
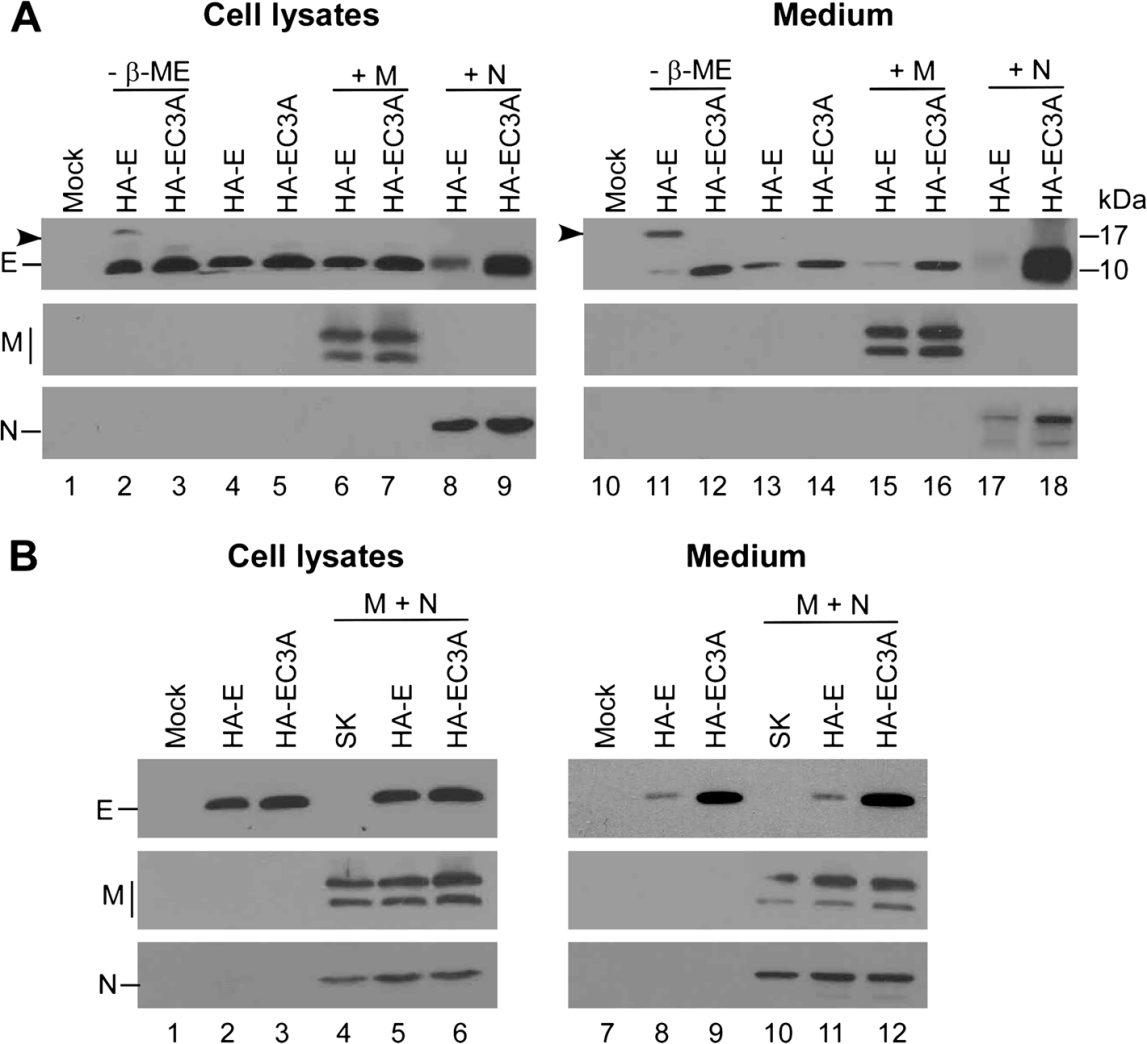
**Changing all three cysteine residues to alanines exerted no negative effects on E release or its capability to interact with N or VLP production. (A-B)** 293 T cells were transfected with SARS-CoV HA-E expression vectors alone, or co-transfected with HA-E plus M and/or N expression vectors. EC3A denotes alanine replacement of all three cysteine residues at E (C40, C43 and C44). Supernatants and cells were collected at 48 h post-transfection and analyzed by Western immunoblotting. Aliquots of cell and supernatant samples containing E proteins (panel **A**, lanes 1 to 3 and 10 to 12) were prepared without reducing reagent (β-mercaptoethanol). SARS-CoV E, M, and N were respectively probed with anti-HA, anti-M and anti-N antibodies. Arrowheads indicate the position of HA-E dimer.

SARS-CoV and MHV E proteins are post-translationally modified with palmitic acid. Lack of palmitoylation modification can markedly decrease SARS-CoV E membrane association [16] and MHV E stability [17]. Substitution mutations for cysteines-the palmitoylation targets in MHV E can reduce virus yields, implying their importance for MHV virus production [14,17]. Accordingly, we tried to determine whether equivalent cysteines play a role in SARS-CoV E/N interaction and VLP assembly by constructing an E mutant (designated EC3A) with alanine replacements for SARS-CoV E cysteine residues C40, C43 and C44. Supernatant and cell lysate samples containing HA-E were analyzed under non-reducing conditions to confirm the presence of disulfide bonds between SARS-CoV E molecules. Results indicate the presence of an 18 kDa band (equivalent to an HA-E dimer) (Figure 4A, lanes 2 and 11, arrowheads), which agrees with a previous report [31]. As expected, only the E monomer was detected in EC3A-containing samples under non-reducing conditions (Figure 4A, lanes 3 and 12).

While most of intracellular E retains its monomeric form, most released E was detected in dimeric form (Figure 4A, lane 2 vs. lane 11), suggesting that released SARS-CoV E proteins are largely linked by disulfide bonds. Interestingly, EC3A was still efficiently released into medium and apparently capable of promoting N release (Figure 4A, lanes 14 and 18). The relatively low level of medium HA-E observed in Figure 4A is due to low expression level (top panels, lanes 17 vs. 8). Nevertheless, our data indicate that EC3A produced VLPs as efficiently as wt when co-expressed with both M and N (Figure 4B, lanes 10–12), suggesting that SARS-CoV E cysteines are not involved in E release, E/N interactions, or VLP assembly. EC3A exhibited greater release efficiency compared to wt, implying that SARS-CoV E cysteines are involved in the E trafficking process. However, immunofluorescence results indicate that the EC3A subcellular distribution pattern was similar to that of the wt.

### Removal of last amino acid residue from the E carboxyl-terminal tail significantly affects E release and E/N interaction

**Figure 5:**
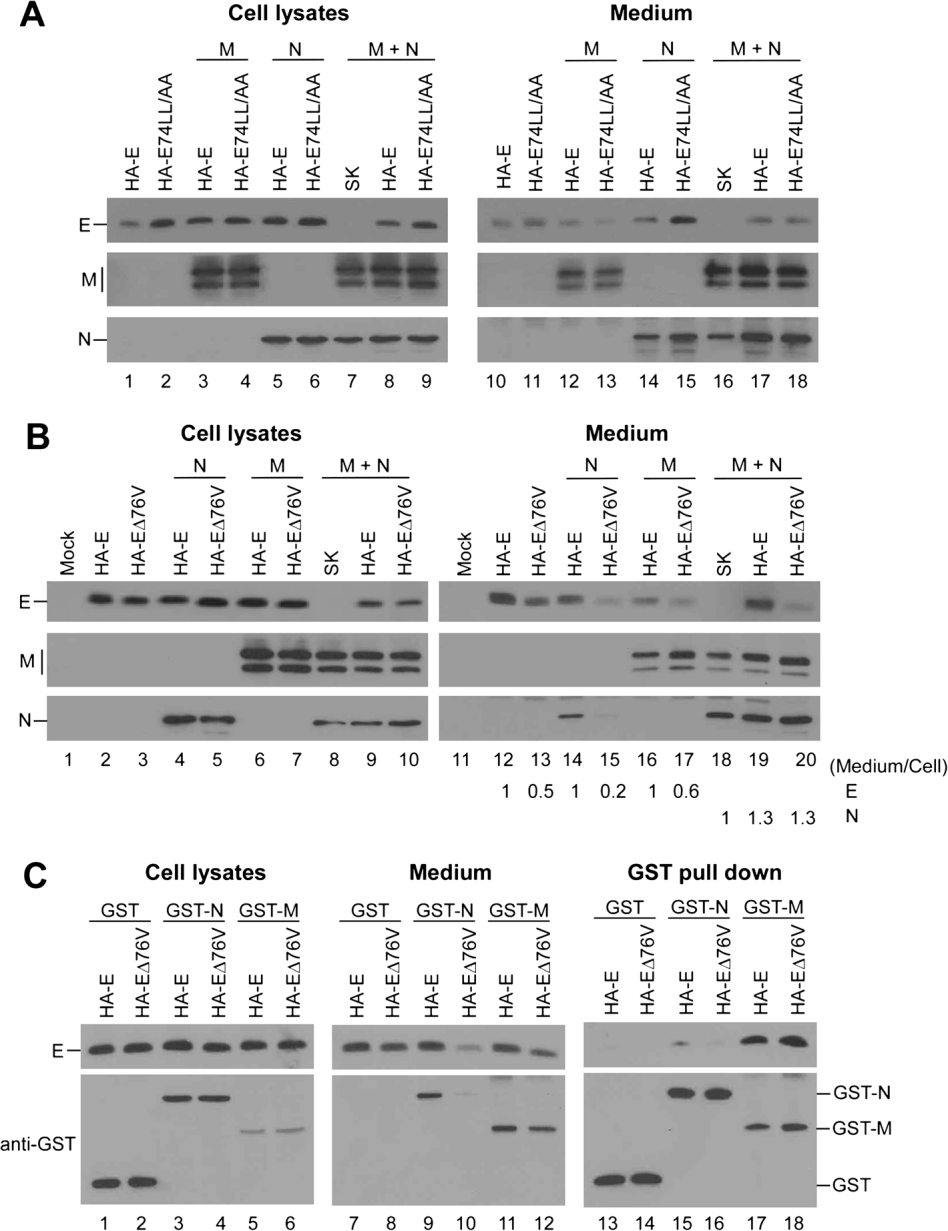
**Effects of E carboxyl tail mutations on E/N association and VLP production. (A-B)** 293 T cells were transfected with HA-tagged E expression vector alone or in combination with M and/or N expression vectors. 74LL/AA indicates alanine substitutions for two leucine residues at the E carboxyl tail (74-LL-75). Δ76V denotes a deletion of the last carboxyl-terminal residue V76 from E. Supernatant and cells were harvested at 48 h post-transfection, prepared, and subjected to Western immunoblotting. E or N proteins from medium or cell samples were quantified by scanning the band densities from immunoblots. Ratios of HA-EΔ76V in media to those in cells were determined for each samples and normalized to that of HA-E in parallel experiment. The level of N-associated VLP (M + N) production is determined as described in the legend to Figure 1. **(C)** Removal of the last E carboxyl-terminal residue significantly affected E/N interaction. 293 T cells were co-transfected with HA-tagged wt or Δ76V and GST, GST-M or GST-N expression vectors. GST-N and GST-M have GST tags at the amino-terminals of the SARS-CoV N and M coding sequences, respectively. At 48 h post-transfection, cells and supernatants were collected and prepared for protein analysis. Aliquots of cell lysate samples were subjected to GST pull down analyses (right-hand panel).

E-FLAG was released at a lower efficiency level compared to HA-E, suggesting that the carboxyl-terminal tail domain may be involved in the E release process. We hypothesized that the E-carboxyl tail is also involved in E/N interaction. To test this idea, we constructed two E mutants: EΔ76V (with the final carboxyl-terminal residue valine removed) and E74LL/AA (with the carboxyl-terminal dileucine motif 74-LL-75 replaced with alanines). According to our results, (a) the E74LL/AA mutant was capable of release, and (b) a positive correlation exists between the release levels of co-expressed N and E74LL/AA (Figure 5A, lanes 10–15). In addition, E74LL/AA was capable of producing VLPs as efficiently as wt when co-expressed with M and N (Figure 5A, lanes 16 vs. 17 and 18). These results suggest that 74-LL-75 was not functionally involved in E release, E/N interaction, or the promotion of VLP production. Conversely, EΔ76A was not capable of efficiently promoting N release, and exhibited a reduced release capacity (Figure 5B, lanes 12 to 15). GST pull-down assay data suggest that the EΔ76V mutation markedly affected E/N interaction, but exerted no major impacts on N/M association (Figure 5C, lanes 16 vs. 18). The level of GST-N-associated E was significantly lower than that of GST-M-associated E, suggesting that E/N association is not as strong as E/M association (Figure 5C, lanes 15 vs. 17). While pull-down assay results suggest an association between EΔ76V and M, EΔ76V release was not significantly enhanced following M co-expression (Figure 5C, lane 18 vs. Figure 5B, lanes 17 and 20). A possible explanation is steric hindrance during VLP assembly. Note that EΔ76V was still capable of promoting VLP production as efficiently as the wt, despite defective packaging into VLPs (Figure 5B, lane 20). This suggests that the SARS-CoV E contribution to VLP production is independent of its ability to be packaged into VLPs.

### None of the mutations significantly affected SARS-CoV E subcellular localization

**Figure 6:**
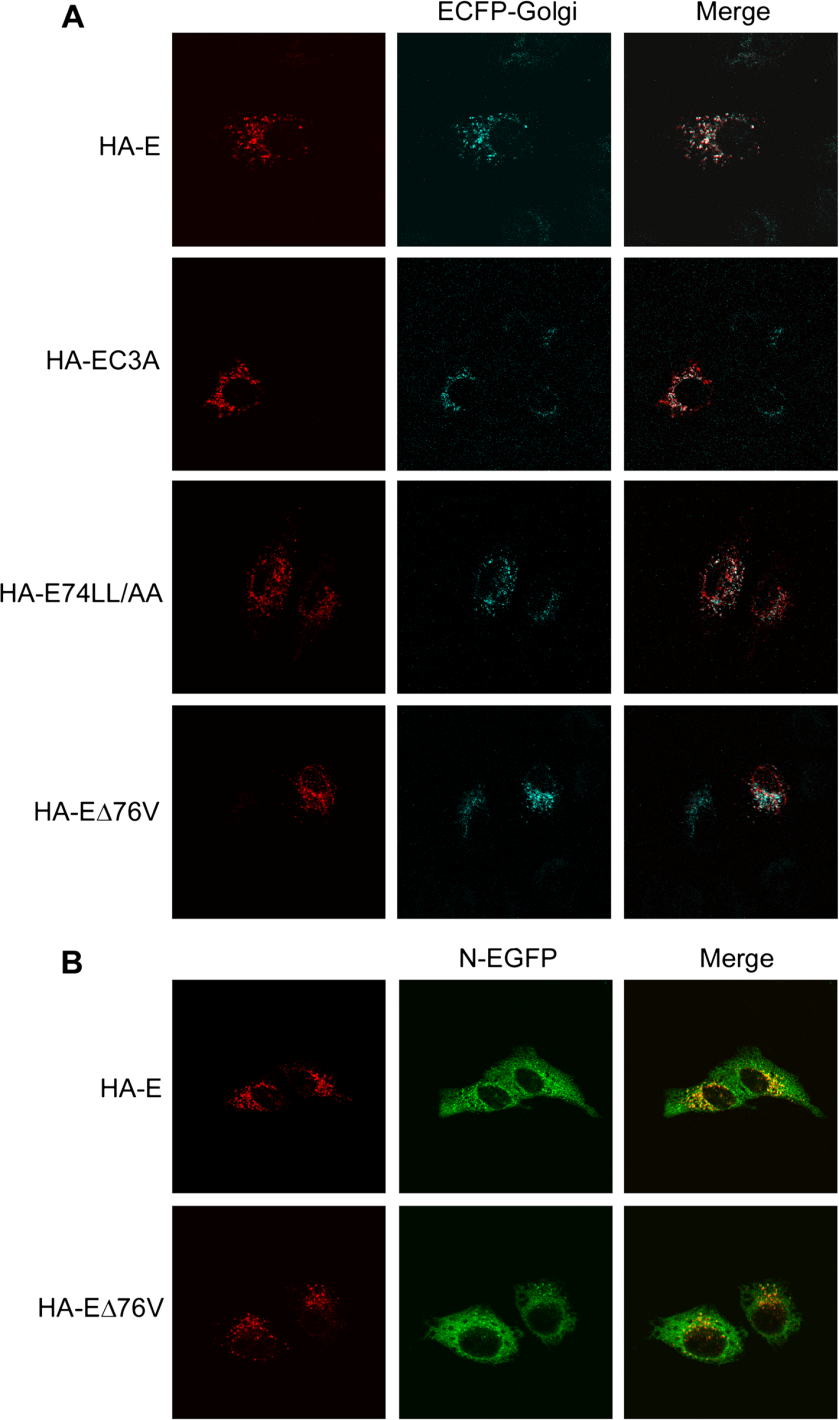
**Subcellular localization of SARS-CoV E and N proteins. (A)** Subcellular localization of wt or mutant SARS-CoV E proteins. HeLa cells were co-transfected with the indicated HA-tagged wt or mutant E expression vectors plus pGolgi-ECFP, which encodes a Golgi apparatus labeling marker. **(B)** Co-localization of SARS-CoV E and N proteins. HeLa cells were co-transfected with SARS-CoV N bearing carboxyl-terminal tagged EGFP (N-EGFP) and HA-tagged wt or Δ76V expression vectors. At 18 h post-transfection, cells were fixed and labeled with a primary anti-HA antibody and a secondary rhodamine-conjugated anti-mouse antibody. Images represent the most prevalent phenotypes. Merged red/blue or red/green fluorescent images are shown in right-hand column panels. Mock-transfected cells failed to yield any signal (data not shown).

To determine whether mutations affecting E subcellular localization also partly accounted for altered release efficiency, we co-expressed each mutant with a Golgi labeling marker (pECFP-Golgi). According to a confocal microscopy analysis, wt and the EC3A, E74LL/AA, and EΔ76V mutants largely localized in the Golgi area (Figure 6A), suggesting that none of the mutations significantly affected E subcellular localization. When co-expressed with EGFP-tagged N, a fraction of N co-localized with EΔ76V in the perinuclear area; this staining pattern was barely distinguishable from that of cells co-expressing HA-E and N-EGFP (Figure 6B). This implies that even though EΔ76V may associate with N to a certain extent, the association is insufficient for enabling EΔ76V co-precipitation with GST-N (Figure 5C).

## Discussion

To our knowledge, this is the first report of interaction between SARS-CoV E and N proteins. Both SARS-CoV spike (S) and E proteins can be released into medium; however, unlike E, S cannot promote the release of co-expressed N (Figure 2A). This suggests that N is incapable of associating with S, and supports the idea of specific E/N interaction. While RNA can enhance N/N interaction, the presence of RNA is not necessary for E/N interaction. Genetic analyses suggest that E binding domains are largely located in the N carboxyl-terminal region (Figure 3).

SARS-CoV M or E by itself can secrete into medium as vesicles but not virus-like particles (VLPs) which can be formed by M plus N, M plus E, E plus N or M plus N and E. It is likely that there may be a combination of these different VLPs in culture medium when cells are co-transfected with M plus E and N. The iodixanol density gradient fractionation analyses suggest that released E vesicles and E-N VLPs exhibit slightly higher densities compared to those of M-E-N VLPs (Figure 2C). Since M is the major viral component, the strong presence of M molecules may exert a spatial effect that explains, at least in part, the lower density for M-E-N particles. We consistently observed marked E-N VLP yield enhancement following M co-expression. Since M is the most abundant viral structural protein capable of recruiting both E and N into VLPs, we do not consider this a surprising result. In addition, M exhibits a noticeably higher N binding capacity than E (Figure 5C). VLPs formed by M, E and N look more morphologically homogeneous than E-N VLPs, possibly due to additional intermolecular interactions between M/M, M/E and M/N.

SARS-CoV E is capable of undergoing oligomerization triggered by both hydrophobic interaction and interchain disulfide bond formation between cysteine residues [16,31]. All three SARS-CoV E cysteine residues have been shown to be post-translationally modified by palmitoylation [16]. Blocking MHV E palmitoylation results in significantly impaired VLP assembly, suggesting that palmitoylated E proteins are essential for murine coronavirus assembly [14]. In the present study we found that changing all three cysteines into alanines (EC3A) did not exert negative effects on SARS-CoV E protein release or VLP assembly (Figure 4). This suggests that E palmitoylation or interchain disulfide bond formation is not required for SARS-CoV E protein release or VLP assembly. Furthermore, we consistently detected higher levels of EC3A compared to wt E in medium, suggesting that EC3A is released more efficiently than wt E. One possible explanation is that post-translational palmitoylation or interchain disulfide bond formation restricts E protein secretion.

Despite a higher release level compared to wt E, EC3A did not produce higher VLP yields than wt E following co-expression with M and N, suggesting no correlation between SARS-CoV E release capacity and its contribution to efficient VLP production. Furthermore, EΔ76A was still capable of enhancing VLP production even though it was defective in both release and N association. Although EΔ76A is capable of M association (as shown by GST assays), it is not efficiently incorporated into VLPs when co-expressed with M. A possible explanation for this discrepancy is that the disruption of cellular compartments allows E/M to associate at a higher capacity, whereas less E/M association occurs in assembly and budding compartments such as ER/Golgi, resulting in smaller amounts of E being packaged into virions. This scenario is compatible with the proposal that E may promote virus assembly and/or budding by facilitating membrane bending and scissions without required packaging into virions. In support of this hypothesis, one research team has reported that E deletion does not significantly affect SARS-CoV replication, but that E-deleted mutants exhibit 100- to 1,000-fold reductions in virus yields associated with decreased efficiency during morphogenesis [32]. Although E/N interactions may not be directly involved in virus assembly and budding, it remains to be seen whether the SARS-CoV E contribution to virus production requires efficient E/M interaction.

## Conclusions

Palmitoylation or interchain disulfide bond formation appears to be dispensable for the SARS-CoV E enhancement of VLP yields. The contribution of SARS-CoV E to efficient VLP production is also independent of its release capacity, association with N, or VLP incorporation. Additional experiments are required to clarify the biological relevance of SARS-CoV E/N interaction, and to determine whether E/N interaction also exists in other coronaviruses.

## Competing interests

The authors declare that they have no competing interests.

## Authors’ contributions

CTW designed the experiments and wrote the paper. YTT, SMW and KJH carried out the experiments and analyzed the data. All authors read and approved the final manuscript.
